# The Thymus Regeneration Paradox: The Search for Stemness in an Involuting Organ

**DOI:** 10.1111/imr.70110

**Published:** 2026-02-15

**Authors:** Roberta Ragazzini, Paola Bonfanti

**Affiliations:** ^1^ Epithelial Stem Cell Biology & Regenerative Medicine Laboratory The Francis Crick Institute London UK; ^2^ Institute of Immunity & Transplantation, Division of Infection & Immunity UCL London UK

**Keywords:** epithelial stem cells, extracellular matrix, in vitro clonal assay, involution and regeneration, thymus, whole‐organ reconstruction

## Abstract

The thymus is emerging as a model for studying organ regeneration and stem cell biology. While research has long focused on how antigen‐presenting cells shape the T cell repertoire, recent discoveries unveil a far richer cellular landscape that challenges long‐held views of thymus structure and function. This review traces the history of early thymic reconstitution assays, the paradigm of clonal stem cells and serial transplantation, assessing evidence for “stemness” within the thymus. A key focus is the paradox that an involuting thymus retains cells able to expand in culture and reconstitute organ function. We differentiate embryonic/fetal thymus development from postnatal homeostasis, emphasizing how the potency of epithelial progenitor/stem cells shifts with age or upon injury. The role of mesenchymal/interstitial cells and the extracellular milieu is considered alongside advances in organ reconstruction. We outline major unsolved questions in the field: thymus regeneration after childhood; the minimal components required to generate functional naïve T cells outside the body; and the potential of next‐generation humanized mouse models to interrogate immune tolerance and novel immunotherapies. We argue that thymus research is entering a new era, one in which understanding and harnessing thymus regenerative potential could yield transformative advances in both basic science and clinical applications.

## 1 The Unpredicted Regeneration of an Involuting Organ

**FIGURE 1:**
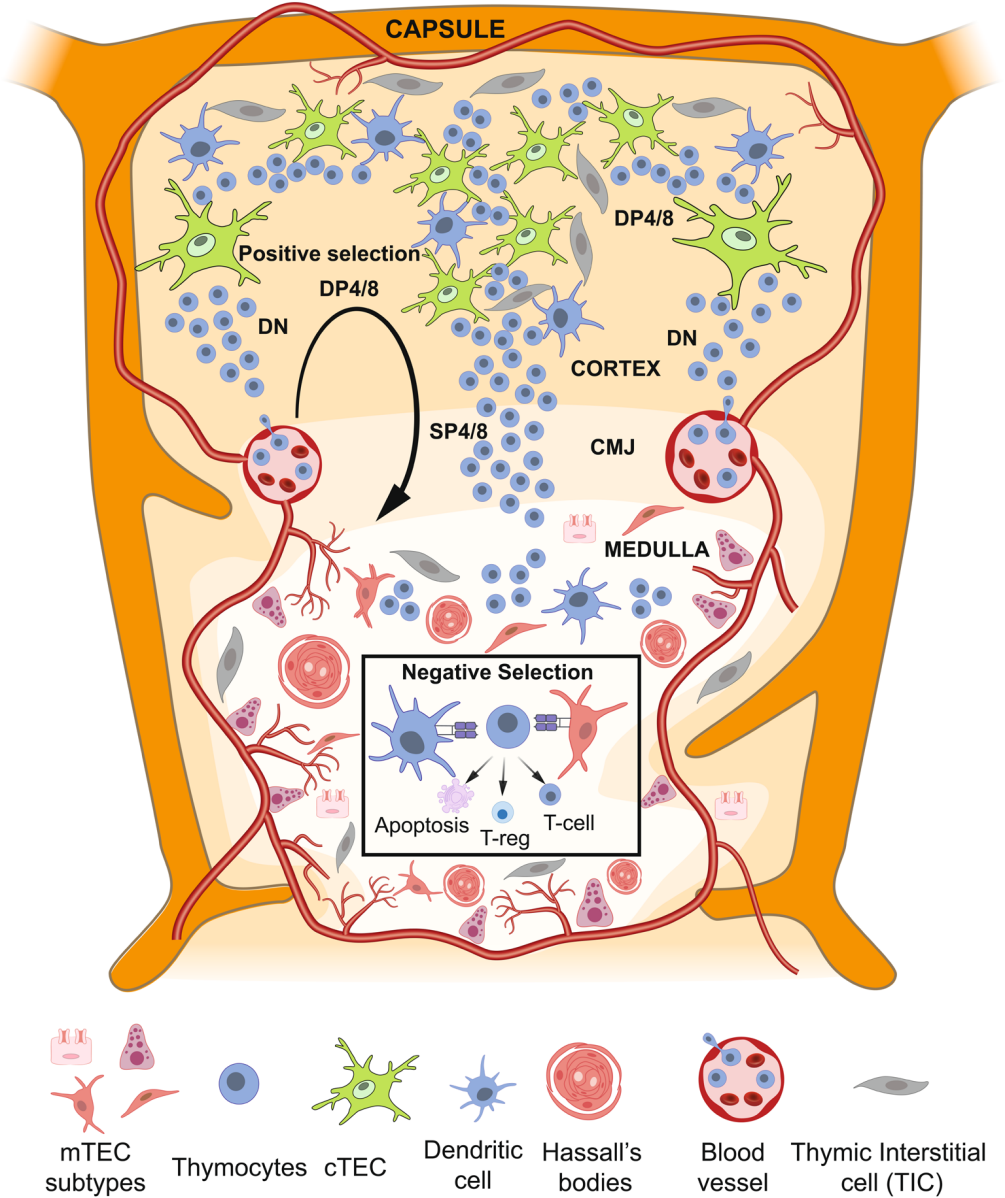
Schematic representing the thymus cellular complexity and functional compartmentalization in cortical and medullary regions. Double‐negative (DN) thymocytes mature in the thymus through different stages, becoming double positive (DP) for CD4 and CD8 molecules and then single positive (SP) CD4^+^ or CD8^+^ T cells. After positive selection by cortical thymic epithelial cells (cTEC), they migrate to the medulla, where they undergo negative selection via deletional tolerance (apoptosis of autoreactive T cells) or dominant tolerance (Treg induction). The human medulla contains Hassall's Bodies (HB), diverse medullary thymic epithelial cell (mTEC) subtypes (e.g., myoid, neuroendocrine, ionocytes), and thymic mesenchymal or interstitial cells (TIC) supporting the thymus architecture. Blood vessels and neural plexus enter through the capsule and interlobular septa.

The thymus is a primary lympho‐epithelial organ, playing a fundamental role in adaptive immunity by safeguarding the body against infections and cancer. It is crucial for the establishment of T cell‐mediated central tolerance, a critical process that strongly reduces the capacity of the immune system to react against the body's own cells, especially against those antigens that are expressed in the thymus. This unique immunological role is mediated by specialized antigen‐presenting cells (APCs), which interact with developing T cells (thymocytes) to determine their immune tolerance or responsiveness to specific antigens (Table 1). Among the thymic APCs are cortical and medullary thymic epithelial cells (cTECs and mTECs, respectively) and dendritic cells (DCs), all of which rely on support from thymic mesenchymal or interstitial cells (TICs). Additional contributors to thymic function include B lymphocytes, endothelial cells, macrophages, and essential structural components such as blood vessels and lymphatic networks (Figure 1).

**TABLE 1:** Key definitions and concepts in thymic antigen presentation and T cell selection.

Antigen presentation and T cell selection in the thymus	
Major histocompatibility complex (MHC) molecules	MHC molecules are cell‐surface glycoproteins encoded by highly polymorphic genes that bind peptides generated by intracellular or endocytic antigen processing pathways and present them to T cells. T cells bind MHC peptide complexes via their antigen receptor. MHC molecules are expressed as class I molecules on the surface of most nucleated cells and class II molecules on specialized antigen presenting cells (APCs). In humans, they are encoded by the HLA locus on chromosome 6.
MHC haplotypes	An MHC haplotype is a defined set of MHC alleles located in *cis* on one chromosome and inherited as a unit from one parent. Because the MHC is highly polymorphic, different haplotypes strongly influence antigen presentation, disease susceptibility and transplant compatibility.
Thymic positive selection	It is a key step in T cell development that occurs in the thymic cortex, where immature T cells, also called thymocytes, interact with MHC‐peptide complexes on cortical thymic epithelial cells. Only thymocytes whose T cell receptors bind MHC‐peptide complexes with low affinity survive and mature into CD4^+^ or CD8^+^ T cells. There is a broad consensus that this process ensures development of a functional, T cell repertoire beacuse thymocytes that are incapable of binding self‐MHC molecules fail to complete the maturation process.
Thymic negative selection	It is a central tolerance mechanism that eliminates developing T cells whose T cell receptors bind with high affinity peptide–MHC complexes. Occurring mainly in the thymic medulla, negative selection is mediated by antigen presenting cells that display MHC molecules complexed with self‐peptides derived from a wide range of tissue‐specific antigens. This process is crucial to prevent autoimmunity by deleting strongly autoreactive T cells. The regulatory T cell lineage is regarded as derived from thymocytes with intermediate affinity for self‐ peptide‐MHC complexes.

The thymus exerts its crucial role during fetal development and early postnatal life. Moreover, it is the first aging organ in our body, displaying a progressive adipogenic atrophy that starts already in childhood; thus, its involution led to thinking that the thymus is useless in postnatal life, considered an “evolutionary accident of no very great significance” [1]. This is also reinforced by the general clinical practice of removing the thymus during cardiac or thoracic surgeries in children, since its location, just in front of the heart, is an obstacle to the access of mediastinal organs after sternotomy. However, the long‐standing belief that early‐life T cell generation provides a sufficient lifelong immunological repertoire is now being challenged by studies demonstrating persistent thymic function in adults [2, 3].

A pivotal observation made several decades ago revealed the presence of residual thymic tissue and immature thymocytes in aged individuals [4, 5]. Furthermore, rebound thymic hyperplasia in adult patients after chemotherapy—that initially provokes an acute thymic atrophy which is followed by regrowth in a high number of documented cases at the end of treatment—is a well‐known phenomenon [6, 7, 8]. Similarly, infections and other physiological stress factors, including pregnancy, are responsible for thymic atrophy that is followed by tissue recovery [9, 10, 11, 12, 13]. Indeed, historical and current models for thymic regeneration that revert its involution typically involve the induction of injury (e.g., through irradiation or chemotherapy) or the ablation of sex hormones by chemical or surgical means [14, 15, 16, 17, 18]. Collectively, clinical and experimental observations have demonstrated that removing these stressors/hormones can trigger thymic regeneration, highlighting the organ's remarkable plasticity and intrinsic capacity for recovery.

These findings suggest that the thymus retains both functional activity and regenerative capacity well into adulthood. While the underlying mechanisms remain elusive, the persistence of thymic tissue suggests an opportunity for targeted therapeutic interventions aimed at rejuvenating immune function, particularly in aging individuals or those with compromised immune systems and chronic disorders such as cancer or autoimmunity.

## 2 Evidence of Stromal Cell Function in Pioneer Thymic Reconstitution Assays

Since the discovery of thymus as a primary lymphoid organ [19], several strategies have been explored to study its “novel” function ex vivo, as the precise cellular mechanisms underpinning thymopoiesis and T cell selection remained unclear. Early efforts to dissect thymic function focused on its developmental stages, as it was clear that its crucial role in establishing immune competence and self‐tolerance is shaped during embryonic and fetal development [19, 20, 21, 22]. Furthermore, the overwhelming abundance of lymphoid cells relative to epithelial and stromal cells poses a significant challenge for the study and isolation of nonlymphoid cellular components at postnatal stages.

**FIGURE 2:**
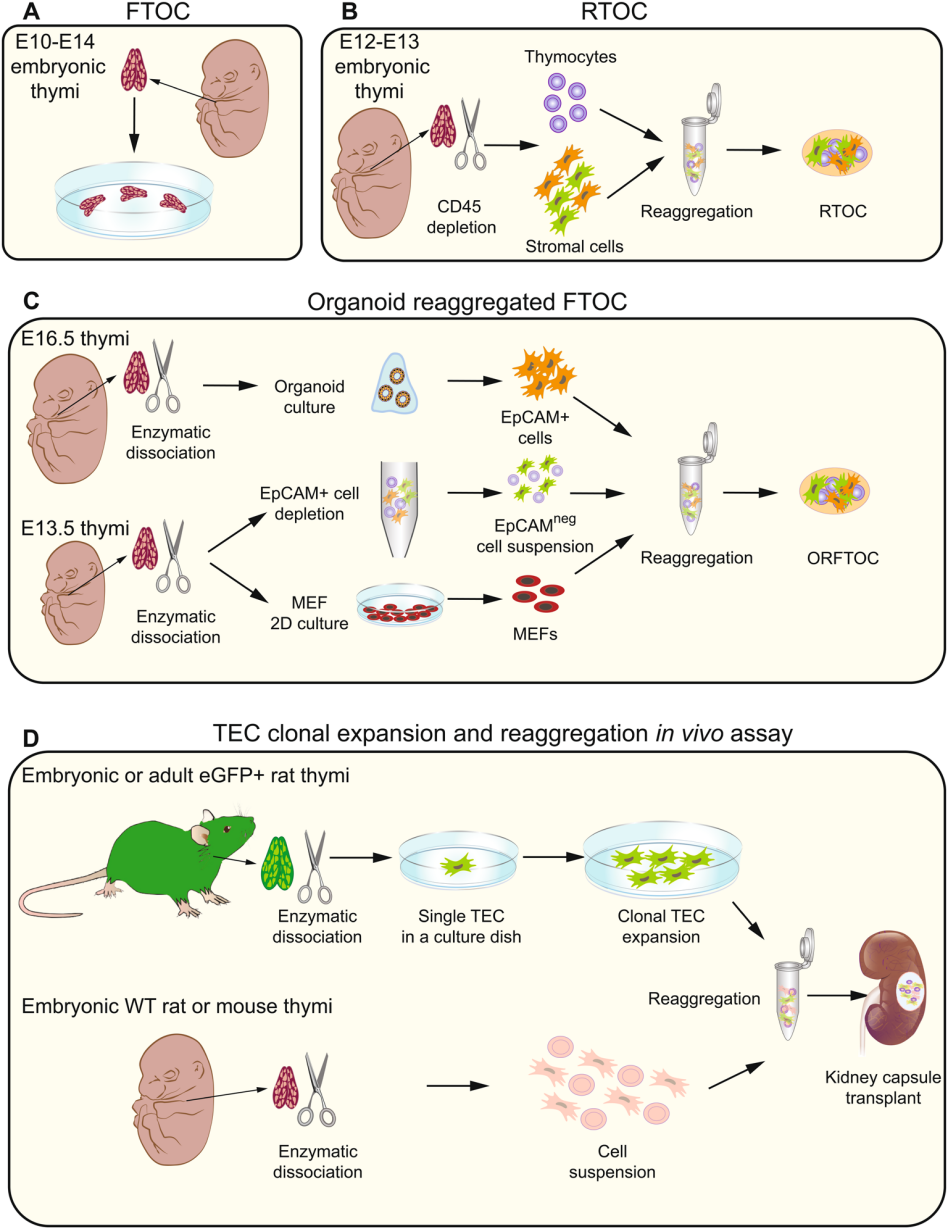
Graphical overview of thymic organ culture and reconstitution assays. (A) Fetal thymic organ culture (FTOC): Isolated embryonic thymic lobes (E10.5‐14.5) are cultured in vitro to study T‐cell development. (B) Reaggregate thymus organ culture (RTOC): Murine embryonic thymi are dissociated into single thymocytes and stromal cells, then reaggregated by centrifugation to investigate cellular interactions within the thymic microenvironment. (C) Organoid‐based reaggregated FTOC: 3D‐expanded EpCAM^+^ thymic epithelial cells (TECs) are reaggregated with EpCAM^−^ E13.5 thymic cells containing T‐cell progenitors to assess TEC function in supporting T‐cell development. Organoids are cultured under conditions promoting TEC proliferation. (D) TEC clonal expansion and reaggregation assay: Developed by Bonfanti et al. [26, 27], this approach demonstrated that both embryonic and adult rat TEC stem cells can self‐renew in vitro and differentiate into functional TECs when reaggregated with embryonic thymic cells and transplanted under the kidney capsule of nude athymic mice, even after serial transplantation.

A key approach to studying the thymus involved culturing the organ in vitro, knowing that embryonic and fetal cells retain the capacity to continue to develop ex vivo [23, 24, 25]. John Owen's team pioneered murine fetal thymus organ culture (FTOC), preserving thymic structure and enabling T cell development (Figure 2A). In FTOC, thymic lobes, extracted from embryonic day 10–14 (E10‐E14) mouse embryos, were maintained under in vitro culture conditions [28, 29, 30, 31]. These studies demonstrated that both stroma and lymphoid cells already present in the thymic anlagen could survive and that T cell development could occur during a culture period of 2 weeks. Moreover, these studies emphasized that microenvironmental factors in vitro, particularly fetal calf serum quality, culture conditions (i.e., pH and temperature), and medium composition, critically influence thymocyte proliferation and differentiation. These findings suggest that T cell differentiation from early lymphoid precursors is an intrinsic, sequential process that can be reproduced in vitro, closely mimicking in vivo development [30]. Moreover, the absence of detection of B cells in these first studies led to the conclusion that the thymus functions exclusively as a site for T lymphopoiesis. Using 20‐deoxyguanosine (dGuo) to eliminate thymocytes while keeping stromal cells intact allowed repopulation with thymocytes from another animal of different haplotypes, thus enabling the first in vitro study on the role of thymus on central tolerance and MHC restriction (Table 1) [32]. Further advances included trans‐filter cultures to repopulate thymic lobes with hematopoietic progenitors, which facilitated lineage‐tracing experiments [33]. The method also helped to analyze stromal‐lymphoid interactions, cytokine effects, and intracellular signaling using knockout mice and retroviral transduction [34, 35, 36]. Importantly, the idea that FTOC correctly recapitulated T‐lymphopoiesis was further supported by the capacity of cultured thymic lobes to continue to grow for several weeks and be repopulated by hematopoietic bone marrow–derived progenitors after transplantation under the kidney capsule of thymectomised neonatal mice, wherein they could reform a thymus organ [29]; this also indirectly confirmed that inflow of hematopoietic progenitors from the bone marrow continues after the very early stages of embryonic development [31]. Thus, FTOC remains a valuable tool in immunology, though it falls short of fully dissecting and reconstructing the functional complexity of the thymus. They had enabled the characterization of T cell development in an in vivo setting, offering important insights into sequential waves of thymopoiesis. However, the highly complex environment orchestrating this process limits the ability to resolve the specific role of individual thymic stromal cell subtypes when studying thymopoiesis and the regenerative potential at postnatal stages.

Further advancements in thymic in vitro models stemmed from the ability to enzymatically break down the extracellular matrix (ECM) releasing the thymic stroma into single cell suspension to isolate TEC and other stromal cells. This enabled the combination of epithelial, stromal, and lymphoid cells in controlled ratios to create reaggregated thymic organ cultures (RTOC) (Figure 2B). Initially, RTOCs were used to study the role of nonepithelial stroma in in vitro thymic cultures [37]. Magnetic bead–based depletion and enrichment techniques facilitated the separation of CD45^+^ lymphoid cells and epithelial cells, which were then recombined through centrifugation, with or without other stromal cells, to confirm TEC's role in T cell development. Murine thymi from embryonic stages E12–E14 when dissociated, and combined with thymocytes in RTOCs, allowed to confirm the critical role of TECs in both positive and negative T cell selection [38]. This pioneering approach, along with improved fluorescence‐activated cell sorting (FACS) methods for isolating specific cell types, paved the way for increasingly accurate models to replicate the thymus' developmental complexity and investigate the function of distinct cell subsets. For instance, this system allowed the validation of chemical genetic systems that regulate biochemical activities such as kinase transgenesis in complex biological processes like lymphoid cell maturation [39]. The duration of in vitro RTOC cultures can range from as short as 2 days to several weeks, depending on the question addressed, although the overall thymic structure cannot be maintained long‐term in vitro as previously discussed [40].

The role of mesenchymal components in thymus development was first highlighted in the 1990s [37, 41, 42, 43], revealing their critical contribution to thymic epithelial architecture and function. However, in the early days, the complexity of thymic stroma and lack of sufficient surface markers to purify epithelial and mesenchymal populations were a big limitation. It is curious to note that even when the RTOC assay showed that mesenchyme was necessary for thymus morphogenesis, it failed to demonstrate a specific role for mesenchymal cells of thymic origin, as thymopoiesis was reconstituted by the use of a NIH‐3 T3 cell line in combination with MHC class II TEC [37]. It is now clear that thymus mesenchymal cells have a crucial role in embryonic and fetal organ development [44]. At this stage, mesenchymal cells help shape the thymic microenvironment, supporting epithelial architecture and providing essential factors to promote cortical TEC proliferation [45] and contribute also to medullary TEC maintenance and regeneration [46]. In adulthood, mesenchymal cells continue to support TEC function by regulating their homeostasis and promoting tissue repair [47] as extensively reviewed in James et al. [48]. Additionally, they play a key role in facilitating T cell development by facilitating thymocyte maturation, survival, and migration through their production of ECM [41]. Importantly, mesenchymal cells also contribute to thymocyte emigration by modulating the ECM and signaling pathways that direct mature T cells into the circulation. Despite their importance, their exact origin and mechanisms of action remain unclear and require further investigation to fully understand their role in postnatal thymus function and maintenance. For example, while the embryonic origin of thymic mesenchyme has been well characterized since the 1970s as deriving from the neural crest [49], the origin of mesenchymal cells in the postnatal and adult thymus remains unknown [50, 51]. For this reason, we refer to human mesenchymal cells in the postnatal and adult thymus as thymic interstitial cells (TICs), using an anatomical designation rather than one based on embryonic origin [52].

In conclusion, the fetal organ culture systems (FTOC and RTOC) offer since decades a valuable tool for investigating T cell subset development and differentiation [53, 54, 55, 56, 57, 58]. The contribution of both systems to the field has been extensively discussed in detail elsewhere [40]. Despite offering valuable insights into thymic physiology, these methodologies are further limited by microenvironmental heterogeneity and reliance on fetal and murine tissue. As a result, they are unsuitable for dissecting the specific roles of individual cellular components in contributing to organ homeostasis and regeneration in postnatal life, and for developing an accurate model of human thymopoiesis.

## 3 The Search for Epithelial Stem/Progenitor Cells in the Thymus

The clear need for advanced methodologies to study the biology of the thymic epithelium beyond development, on one hand, and its impact on thymopoiesis, on the other, had driven the search for cells and mechanisms responsible for tissue homeostasis and regeneration. In many tissues, especially those that undergo constant renewal, such as blood, epidermis, and the gut, maintaining integrity and allowing repair and regeneration rely on the presence of long‐lasting, self‐renewing stem cells (SCs) that adapt and interact with a specific microenvironment or “niche” [59, 60]. Similarly, changes in stem cell properties and their niches with aging remain one of the most debated topics in regenerative medicine [61].

In the 1950s, adult hematopoietic stem cells (HSCs) were the first identified stem cells, defined by their capacity for clonal growth and multilineage fate potency upon radiation injury [62, 63]. In the following decades, HSCs were further characterized and later isolated based on the expression of specific surface marker combinations [64, 65, 66, 67]. This approach sparked broader interest in the search for adult stem cells in other tissues and organs, including the thymus. What emerged was a major debate with multiple, often conflicting studies identifying various putative epithelial progenitors, each defined by distinct markers and functional properties [68]. These findings added difficulty to the understanding of thymic epithelial heterogeneity and its origin. One of the earliest progenitor populations was identified by the expression of the surface glycoprotein MTS24 in Keratin‐(KRT)5 and KRT8 double‐positive epithelial cells [69, 70]. Although this rare population persisted in the adult murine cortex and medulla, only embryonic MTS24‐positive cells showed the ability to differentiate and support the development of mature T cells in RTOCs and upon in vivo transplantation [70]. Moreover, MTS24‐positive cells expressed placenta‐expressed transcript (Plet‐1), a marker associated with progenitor cells in other epithelia. However, in the thymus, Plet‐1 expression is restricted to early embryonic progenitors and is not preserved across species [71].

In the quest to identify optimal surface markers for thymic progenitors, studies utilizing reporter and transgenic mouse models provided evidence of a bipotent epithelial progenitor capable of giving rise to both cortical and medullary TECs [72, 73]. A clonal assay was first employed to assess the developmental potential of single, individually selected TECs that constitutively expressed enhanced yellow fluorescent reporter protein (eYFP). Embryonic eYFP‐positive mouse TECs were dissociated to a single‐cell suspension and microinjected into wild‐type embryonic thymic lobes. These single cells successfully integrated into the thymic architecture and differentiated into both cortical and medullary TECs, thereby providing further evidence to the endoderm‐only origin of TECs [72]. However, these studies primarily focused on the developmental potential of embryonic progenitors, without addressing their role in postnatal organ maintenance or regeneration.

Efforts to identify putative postnatal progenitors in the mouse thymus involved isolating cells based on different combinations of surface markers previously reported in the thymus or other epithelial tissues. The group of Ann Chidgey and Richard Boyd used major histocompatibility complex II, proposing that low expression levels (MHCII^low^) mark less functional or less differentiated TECs. They further selected MHCII^low^ cells co‐expressing α6‐integrin (CD49f^high^) and stem cell antigen‐1 (Sca‐1^high^) [74]. Meanwhile, the group of Clare Blackburn isolated cells co‐expressing Plet‐1 and Ly‐51 [75]. All these FACS‐sorted populations were then re‐aggregated with dissociated embryonic thymic lobes to reconstitute hybrid RTOC, which were transplanted in vivo under the kidney capsule of nude or syngeneic mice. The persistence of these cells in vivo for 4–12 weeks up to 9 months suggested their progenitor nature; however, this approach did not formally demonstrate their self‐renewal capacity, as embryonic developmental cells and cues within RTOC were still driving and supporting TEC survival and differentiation.

To assess in vitro growth potential, Wong et al. [74] employed a 3D in vitro system based on Matrigel, where TEC subsets isolated from Foxn1^eGFP/+^ reporter mice (aged 6–8 weeks) were cultured alongside mouse embryonic fibroblasts. Under these conditions, Foxn1^eGFP/+^ putative progenitors were phenotypically characterized after 1 week in culture, but they progressively lost the ability to form new 3D colonies and exhibited reduced growth potential upon sub‐culturing [74]. Another in vitro approach to characterize epithelial progenitors took advantage of the ability of some TECs to form spheroid colonies under low‐attachment culture conditions in the presence of specific growth factors, an approach originally developed for neuronal and mammary progenitors [76, 77]. Ucar et al. used this system to generate “thymospheres” from the CD45‐negative fraction of adult murine thymus. These CD45‐negative thymospheres were also EpCAM‐negative and Sca1‐positive, highlighting the important observation that not all mouse TECs are EpCAM‐positive in culture. While the organoid field based on Matrigel 3D cultures emerged with Lgr5‐expressing intestinal stem cells which are fast cycling, thymospheres are slow cycling in vitro and display high heterogeneity [78]. They exhibit cells of both cortical and medullary features, but their long‐term self‐renewal in vitro remains unexplored. Notably, thymosphere formation does not require *FoxN1* gene expression, challenging the notion that bipotent progenitors depend on and are identified by the presence of FoxN1, the key transcription factor for thymus development [78].

Only very recently, organoid cultures have been adapted to expand murine TECs [79, 80, 81]. EpCAM, as a pan‐epithelial marker, was used to isolate TECs which were then seeded in 3D Matrigel or equivalent, as well as in a microwell‐array miniaturized organoid culture in polyethylene glycol (PEG)–based hydrogel. Most frequently, thymic organoids are derived from embryonic and newborn (P0) mice [80, 81]. These cultures were characterized only during the first week, up to day 7 (D7), during which they retain some TEC identity features but do not express functional markers. For functional validation, these expanded organoids were reaggregated in FTOC (named ORFTOC) with EpCAM‐depleted E13.5 thymic lobes, which supported thymocyte development to single‐ and double‐positive (CD4^+^CD8^+^) T cells (Figure 2C). However, the presence of various stromal and hematopoietic embryonic cells in these reaggregates means that the potential contribution of other stromal populations cannot be ruled out, particularly in the presence of embryonic cues [80]. In another approach, TECs isolated from dissociated thymi of 6‐ to 12‐week‐old mice were directly cultured in 3D organoid systems for several passages [79]. The addition of the well‐known mesenchyme‐derived growth factor FGF7 (a.k.a. keratinocyte growth factor, KGF) [82, 83, 84] proved particularly important for sustaining organoid expansion. These expanded organoids were further induced to differentiate into more mature cortical and medullary subtypes through signals provided by exogenous molecules such as retinoic acid (RA) and RANKL, as well as co‐culture with freshly isolated thymocytes [79]. Within this system, thymocyte maturation occurred from double‐negative (DN) cells, albeit with low efficiency toward the CD4 lineage, likely due to low MHC class II expression and the absence of key signals required for physiological thymopoiesis. While TECs were reported to expand long‐term and differentiate, the characterization of TEC progenitor cells within this in vitro organoid model remains incomplete as well as the capacity to fully support thymopoiesis [79].

Overall, these studies advanced the knowledge of TEC biology in animal models and provided evidence for epithelial progenitor cells capable of differentiating into cortical and medullary compartments. However, most research has focused on embryonic development, often relying on reaggregation assays in which putative epithelial progenitors, freshly isolated from mice of different ages, were still influenced by an embryonic microenvironment or its cues (Table 2). Indeed, murine epithelial cells are notoriously difficult to establish 2D cultures without immortalization [86, 87, 88]. The development of 3D organoid in vitro systems for TECs enabled in vitro expansion followed by differentiation of murine putative progenitors on a small, limited scale. Yet, these studies still lacked robust quantitative tools and functional assays to identify *bona fide* stem cells in the postnatal and adult thymus, equivalent to the well‐established clonal analysis and serial transplantation used to define the “professional” stem cells in other adult tissues, such as HSCs and epidermal keratinocyte or corneal stem cells [67, 87, 89, 90, 91, 92].

**TABLE 2:** Synoptic table summarizing the key studies investigating the existence, potency and function of thymic epithelial progenitor or stem cells in the mammalian thymus (mouse, rat, and human).

Source of thymic epithelial stem or progenitor cells (species and donor age)	Gene/protein expression defining epithelial stem or progenitor cells	In vitro expansion conditions	Assay(s) for demonstrating self‐renewal and functional differentiation	Limitations	Original references
Murine thymus embryonic stage E12	eYFP reporter; EpCAM, KRT5, KRT8	na	Freshly isolated eYFP+ progenitors were injected as single cells into E12 wt thymic lobules and transplanted under the kidney capsule of nude mice for 4 weeks. Both cTEC and mTEC were obtained by eYFP+ cells, thus proving thymic E12 TEC are bipotent progenitors	Use of only embryonic progenitors; TEC differentiation is driven by an embryonic‐developmental microenvironment; no proof of capacity of eYFP+ TEC to support T cell development.	Rossi et al. [72], PMID: 16791197
Murine embryonic thymus: in vivo lineage tracing analysis induced at embryonic age	eYFP label, EpCAM, KRT5, KRT8	na	Lineage tracing experiments in vivo to prove differentiation of embryonic progenitors in cTEC and mTEC; E12.5 MHC‐classII+eYFP+ TEC were sorted, reaggregated with E14.5 thymic lobes of wild‐type mice and then transplanted under the kidney capsule of nude mice for 20 weeks to confirm cTEC and mTEC fate potency.	Use of embryonic progenitors; TEC differentiation is driven by an embryonic/developmental microenvironment; low labeling efficiency in KRT14 lineage traced model; no proof of capacity of YFP+ TEC to support T cell development.	Bleul et al. [73], PMID: 16791198
Murine thymus embryonic stage E11.5	Plet‐1, MTS20, MTS24	na	na	Description of embryonic progenitors; lack of differentiation and functional assays.	Depreter et al. [71], PMID: 18195351
Rat thymus embryonic (E16); postnatal day 1 (P1), and 4‐month old (4mo)	eGFP reporter; EpCAM, KRT5, KRT8, TP63 (TEC multipotent stem cells)	Single cell clonal assay in 2D and extensive clonal expansion of stem cells.	Rat eGFP+ TEC clonally expanded and reaggreagated with E14.5 wt mouse thymic cells differentiate into cTEC and mTEC expressing functional markers (e.g., Aire, MHCII). Fusion was excluded by FISH for rat and mouse probes both in vivo and in vitro, upon recovery in culture after transplantation. Clonally expanded eGFP+ TECs proved stemness and multilineage potency upon serial transplantation like hair‐follicle multipotent stem cells in the skin.	TEC differentiation within a mouse‐rat chimeric microenvironment, with no direct proof of T cell development directly supported by rat eGFP+ TEC.	Bonfanti et al. [26], PMID: 20725041
Murine thymus 6–8‐week‐old	ItgA6, Sca‐1, MHC‐II low	3D culture in 50% Matrigel with MEF; clonal assay.	Sorted TEC subsets reaggregated with dissociated thymi from E14.5 mice and transplanted under the kidney capsule of nude mice for 4–12 weeks to assess TEC differentiation and thymopoiesis.	Absence of long‐term expansion of TEC progenitors in vitro; TEC differentiation is driven by an embryonic/developmental microenvironment; no direct proof of supporting T cell development.	Wong et al. [74], PMID: 25131206
Murine thymus 4–6‐weeks‐old	EpCAMnegative, Sca‐1, CD24negative, Foxn1negative	Thymosphere suspension expanded up to 4 passages and on collagen surface for 9 days.	Thymosphere generation with spontaneous differentiation in vitro; label retention assay of thymosphere in vitro; thymospheres were reaggregated with dissociated thymi from E14.5 mice and transplanted under the kidney capsule of nude mice for 4–6 weeks to confirm differentiation potential in vivo.	Absence of long‐term expansion of thymospheres in vitro and monolayer cultures; TEC differentiation is driven by an embryonic/developmental microenvironment; no direct proof of T cell development in vivo.	Ucar et al. [78], PMID: 25148026
Murine thymus 8‐week‐old	Plet‐1, Ly‐51, MHCII‐high (TEC progenitors re‐aggreated with embryonic thymi)	na	Adult TECs were sorted, grown by limiting dilutions and reaggregated with unfractionated dissociated E12.5 or E13.5 thymic cells and mouse embryonic fibroblasts (MEFs) at defined ratios for 12–16 h in vitro, then transplanted under the kidney capsule of syngeneic recipient mice for 4 weeks to 9 months to prove their differentiation potential in vivo.	No evidence for self‐renewal capacity of isolated progenitor population; TEC differentiation is driven by an embryonic/developmental microenvironment; no direct proof of T cell development in vivo.	Ulyanchenko et al. [75], PMID: 26997270
Human thymus postnatal ages: 0–17‐year‐old.	KRTs, BCAM, FN1, IFITM3, TIMP1, CLU, CD90, ITGA6 (PolyKRT multipotent stem cells)	Single cell clonal assay in 2D; extensive expansion of stem cells upon passages; in vitro differentiation in serum free medium.	Cultured clonal PolyKRT stem cells (i) retain cortex/medulla self‐organizing capacity in vitro in absence of other cell type/microenvironmental cue; (ii) are capable of differentiating into functional cTEC/mTEC in vivo which attract lymphoid progenitors from humanized bone marrow and (iii) support their maturation to functional T cells in athymic NSG‐FoxN1null mice. First human whole‐organ reconstruction from cultured somatic stem cells.	Human model system is limited by the absence of lineage tracing experiments in vivo.	Ragazzini et al. [85], PMID: 37652013
Murine thymus 6–12‐week‐old	EpCAM, Krt5, Krt14, Sox15, Trp63 (organoid culture)	Expansion of organoids up to 2 years, clonal expansion.	TEC organoids are co‐culture with freshly sorted double negative (DN) thymocytes to prove TEC differentiation and T cell development in vitro; co‐cultured organoids plus DN were transplanted subcutaneously into Nude mice for 7 weeks to prove in vivo thymopoiesis.	Lack of characterization of thymic progenitors, no expansion in large numbers in matrigel; T cell development in vitro starting from DN precursors with a faster timeline; low efficiency T cell differentiation lacking also double positive in vivo; low yield of CD4 was obtained due to low MHC II expression.	Lim S et al. [79], PMID: 38551965
Murine thymus embryonic stage E16.5 and postnatal day 0 (P0)	EpCAM, Krt5 and Krt8 (organoid culture)	Expansion of organoid in serum free medium plus FGF‐7.	In vitro culture of ORFTOC obtained by reaggregating E13.5 EpCAM‐neg MEF with disaggregated organoids to prove their differentiation potency and thymopoieisis; ORFTOC transplanted under the kidney capsule of syngeneic CD45.1 recipient mice for 5 weeks to prove organoid TEC can attract bone marrow progenitors and support thymopoiesis.	Use of embryonic TEC, lack of characterization of thymic progenitors, lack of in vitro clonal assay and long‐term expansion; T cell development in vitro is from embryonic progenitors with a faster timeline; syngeneic mice have an endogenous thymus with confounding functional readout in vivo.	Hubscher et al. [80], PMID: 39036995

*Note:* Columns provide details on the tissue donor species and age; gene marker profile for cell identification and/or isolation; in vitro culture conditions; experimental assays to demonstrate progenitor or stemness nature; and limitations of each study. Row colors indicate donor cell species: Light color for mouse; medium for rat; dark for human.

Abbreviations: Aire, auto immune regulator; BCAM, basal cell adhesion molecule; CLU, clusterin; eGFP, enhanced green fluorescent protein; EpCAM, epithelial cell adhesion molecule; eYFP, enhanced yellow fluorescent protein; FGF‐7, fibroblast growth factor 7; FISH, fluorescence in situ hybridization; FN1, fibronectin1; IFITM3, InterFeron‐Induced TransMembrane protein; Itga6/ITGA6, Integrin Alpha 6; Krt/KRT, keratin; MEF, mouse embryonic fibroblats; MHC‐II, major histocompatibility complex class II; ORFTOC, organoids reaggregated fetal thymus organ cultures; Plet‐1, placenta expressed transcript 1; Sca‐1, stem cells antigen‐1; Sox15, SRY‐box Transcription Factor 15; TIMP1, tissue inhibitor of metalloproteinase 1.

## 4 Applying the Paradigm of Clonal Stem Cells and Serial Transplantation to Thymic Epithelium

The fundamental property of stem cells to self‐renew in vivo, ensuring lifelong functional renewal and repair of tissues and organs, was experimentally defined through the serial transplantation assay developed by Till and McCulloch for bone marrow HSCs [92]. The capacity to transplant HSCs from a donor to a recipient and subsequently recover and re‐transplant them into another host demonstrated that stem cells can outlive the organism from which they were originally derived. The experimental proof that stem cells can also self‐renew in vitro, retaining their full differentiation potency, was provided by Claudinot et al. [87] using a rat model which constitutively expressed enhanced green fluorescent protein (eGFP) in every cell. Their work investigated the long‐term self‐renewal capacity of cultured epithelial stem cells in rodents by combining single‐cell clonal assay in vitro and serial transplantation of eGFP‐labeled hair follicle stem cells [87]. Their findings confirmed the multipotency of hair follicle stem cells, demonstrating their ability to generate diverse cell lineages, including epidermis, sebaceous glands, and hair follicles, and outlive the organism from which they were derived for several years in culture [87].

Moved by the observation that within the thymus some medullary cells share gene expression profile and give rise to stratified structures (Hassall's bodies) typical of a multilayer squamous epithelium such as the one of the skin, rodent TECs were challenged for their capacity to grow at clonal level and self‐renew in vivo and in vitro [26]. Using the same eGFP rat model as in Claudinot et al., thymi of different embryonic and adult ages, including E16, P1, and 4‐month‐old, were dissociated to derive 2D cultures (Figure 2D). TECs grew forming round colonies and expanded robustly as in the original culture method reported for human skin keratinocytes [93]. It is important to highlight that this same culture system does not allow robust expansion of mouse epithelial cells [86]. Instead, rat TECs could be grown as clonal cells, being individually seeded into a 35‐mm petri dish. The progeny of each growing clone gave rise to millions of cells demonstrating long‐term expansion and self‐renewal capacity for more than 13 passages without any sign of senescence and maintaining a stable karyotype. eGFP^+^ rat TECs retain the expression of key genes essential for TEC identity and exhibit a keratin profile indicative of both cortical and medullary origins. Remarkably, when these cultured TECs are aggregated with freshly isolated thymic cells and transplanted in vivo under the kidney capsule of nude athymic mice, they contribute to thymic morphogenesis. The resulting structure not only mimics the three‐dimensional architecture of the native thymus but also demonstrates functional properties, such as re‐expression of MHC class II and AIRE (autoimmune regulator), and the presence of both CD4^+^ and CD8^+^ single‐ and double‐positive thymocytes (Figure 2D) [26].

The ability of these clonogenic TECs to rebuild a functional thymic structure suggests they may act as thymic epithelial stem cells. In a key experiment, when these clonogenic TECs were placed beneath the surface layers of developing mouse skin, where they were exposed to skin‐specific signals, they contributed to the formation of both the epidermis, sebaceous glands, and hair follicles. Even more importantly, serial transplantation experiments showed that these TECs could sustain long‐term renewal also of epidermis, something that native hair follicle stem cells are unable to do beyond a few weeks (tissue turnover) or in response to injury [26, 27, 87].

Further analysis of eGFP^+^ rat TECs revealed that once they adapt to the skin environment and are re‐isolated for subsequent culture, they undergo a stable shift in their gene expression profile. This transcriptomic reprogramming appears to be lasting and does not change further, even after multiple rounds of culture and transplantation, suggesting that a single environmental cue is enough to redirect their fate. Interestingly, unlike typical skin stem cells, these reprogrammed TECs retain the unique ability to return to the thymus and reintegrate into its architecture [26, 27]. Perhaps most compelling is the demonstration that a single TEC, once exposed to the skin environment, can later contribute to both skin and thymic tissue [27]. These findings reveal an unexpected degree of plasticity: clonogenic TECs can function not only in thymic regeneration but also as multipotent stem cells capable of supporting epidermis, sebaceous gland, and hair follicle development. What drives this remarkable plasticity, and how these cells react to such distinct environmental signals, remain open and exciting questions.

Together, these results reveal that the rodent thymus harbors epithelial stem cells capable of expanding in vitro and fulfilling the key functional and molecular criteria of epithelial stem cells, similar to those found in the skin. This challenges the traditional view of TECs as lineage‐restricted and instead highlights their capacity for broader developmental potential. The ability of TECs to respond to environmental cues and undergo stable reprogramming into entirely different epithelial lineages underscores a previously underappreciated level of cellular plasticity [94]. A central question is whether this potential and intrinsic feature of thymic epithelial stem cells is at the origin of the high heterogeneity of TEC populations of the adult thymus, a concept that has gained growing evidence in recent years thanks to novel technologies. Understanding the molecular basis of this plasticity is a critical avenue for future research, with far‐reaching implications for regenerative medicine, immune reconstitution, and epithelial biology.

## 5 Stemness Within the Human Thymus

**FIGURE 3:**
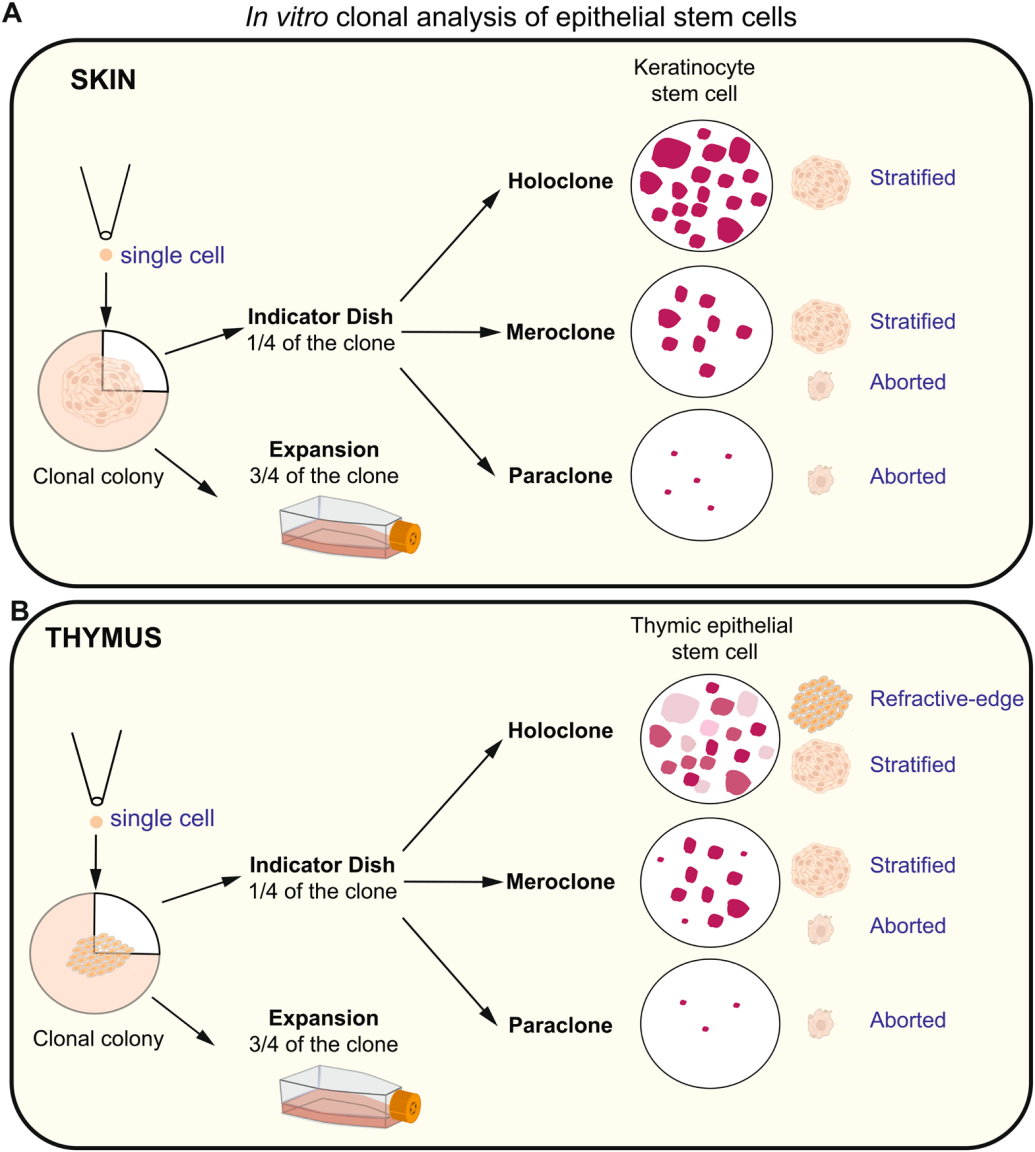
In vitro single‐cell clonal assays for human epidermal keratinocyte and thymic stem cells. (A) Clonal assays trace the fate of individual cells and their progeny, discriminating short‐lived progenitors from long‐lived stem cells. In the 1980s, Barrandon and Green pioneered this method by plating single epidermal cells and tracking colony formation [97]. Clones were partially replated, cultured for 12 days, and stained with Rhodamine‐B (a.k.a. Indicator Dish). Based on morphology and proliferative capacity, three clone types were identified: Holoclones (highly proliferative, stem cell), meroclones (intermediate, transient amplifying progenitor), and paraclones (limited potential, aborted colonies). (B) More recently, Ragazzini et al. [85] applied this assay to thymic epithelial cells in vitro, identifying holoclone‐like colonies with refractive edges along with meroclone and paraclones with stratified morphology.

The advent of epithelial stem cell culture method in the 1980s marked a turning point in regenerative medicine, thanks to the pioneering work of Howard Green. His groundbreaking development of epidermal transplants has saved thousands of lives, particularly in patients with severe and extensive burns [95, 96]. The single‐cell clonal assay developed by Yann Barrandon in Green's laboratory provided the functional definition of *bona fide* epithelial stem cells in a dish (Figure 3A) [97]. This assay demonstrated that certain epithelial cells can self‐renew in vitro long‐term while retaining this ability in vivo after culture. Only cells capable of generating long‐lived, expanding colonies (termed “holoclones”) were found to be both necessary and sufficient to ensure lifelong renewal of the epithelium [97, 98, 99]. This breakthrough established the foundation for durable, stem cell–based therapies across a range of medical conditions [89, 98, 100, 101, 102]. More recently, this approach has been applied to treating congenital diseases where cultured stem cells are combined with gene therapy to treat previously incurable diseases [103, 104]. These advances highlight the importance of defining the properties of clonogenic stem cells, particularly their ability to extensively expand in vitro for successful clinical translation and long‐term therapeutic efficacy.

In rapidly renewing organs like the skin or gut that renew their epithelium completely every 3 weeks or 4 days, respectively, the presence of actively proliferating stem cells is expected. But what about the thymus, an organ that begins to shrink and lose function progressively soon after birth, eventually becoming a soft, fatty remnant in adulthood and old age? Why would such an organ, seemingly destined to involution, possess cells with self‐renewal capacity or the potential to grow extensively in vitro? Motivated by rodent studies showing that TECs have clonogenic potential and remarkable plasticity (as discussed above) [26, 94], we set out to investigate whether similar “stemness” properties could characterize a subset of TECs in the human thymus.

Recent advances in single‐cell RNA sequencing (scRNA‐seq) have enabled detailed characterization of the transcriptomic landscape of the murine and human thymus, revealing high heterogeneity among TECs at unprecedented molecular resolution [52, 105, 106, 107, 108, 109]. However, these studies have been limited by a lack of epithelial cell subtype enrichment and by isolation strategies that did not account for the low abundance TECs (< 0.02%) within an organ mainly populated by thymocytes (> 99%), thus reducing the resolution of TEC subtypes described. To overcome these challenges, successive rounds of stromal enrichment were employed, followed by independent scRNA‐seq analyses of the cortical and medullary epithelial cells, sorted as EpCAM^low^CD205^+^ (cTECs) and EpCAM^high^CD205^−^ (mTECs), respectively [85]. This approach revealed previously unrecognized cellular heterogeneity in both compartments by describing new specialized mTEC and cTEC subtypes (e.g., cTEC‐progenitors, cTEC‐IEGs, mTEC‐progenitors, mTEC‐Mechanosensory). Most importantly, TECs isolated from both cortex and medulla defined a distinct and similar cluster suggestive of representing a putative epithelial stem cell population. In fact, these cells do not express any medulla or cortex specialized signature and instead express marker genes characteristic of epithelial stem cells in other tissues, including *CEBPD, CLU*, and *LIFR* [110, 111, 112] as well as genes involved in antiviral responses such as *CH25H* and *IFITM3*, which are known to confer viral resistance in various tissue‐resident stem cells [113].

Unexpectedly, this epithelial population exhibits an unusual co‐expression of multiple keratins typically associated with distinct epithelial lineages, including *KRT15*, a marker of proliferative basal epithelial cells, and *KRT13*, found in suprabasal, terminally differentiated layers in stratified epithelia [114]. In addition, these cells express keratins characteristic of both simple epithelia (e.g., *KRT8, KRT19*) and of stratified epithelia (e.g., *KRT5* and *KRT14*) [115]. Due to this broad although specific keratin expression profile, the population was termed polykeratin (polyKRT) [85]. This atypical keratin co‐expression may reflect the inherent plasticity of thymic epithelial stem cells, a feature previously observed in rodent clonogenic TECs following heterotopic transplantation into the skin [26] (Table 2).

A subset of this cluster expresses proliferation‐associated genes, including *ANLN, AURKB*, and *CCNA2*, which are part of the skin keratinocyte holoclone transcriptional signature [116]. Based on this profile, they were designated as polyKRT‐proliferating cells [85].

The high resolution of this scRNA‐seq dataset further enabled the identification of transitional epithelial clusters linking polyKRT cells to differentiated cortical and medullary TECs, defining the presence of cortical and medullary lineage‐restricted progenitors in the human thymus. Among these, a progenitor population upstream of cortical TEC is characterized by expression of *ATF3* and *KCNIP3*, whereas a progenitor population upstream of medullary subtypes is characterized by *CLDN3* and *CLDN4*, reminiscent of medullary progenitors described in murine models [117, 118]. These mTEC progenitors also express *ASCL1*, a pioneer transcription factor essential for the development of neuronal and neuroendocrine lineages [119, 120, 121, 122]. These findings support a hierarchical model in which human TEC differentiation proceeds through defined progenitor intermediates with lineage bias and distinct transcriptional programmes which was supported by scRNA‐seq trajectory analysis [85].

Although in silico technologies are extremely helpful for generating hypotheses related to cell identity and developmental potency, their predictive power is limited by current knowledge and methodological variability. Discrepancies across studies often arise from differences in original tissue quality, sequencing depth, and computational pipelines. Therefore, wet lab–based experimental validation is essential to substantiate computational inferences and to move beyond trajectory‐based predictions or mathematical modeling. For instance, single‐cell RNA‐seq can aid in identifying novel candidate marker genes and molecules associated with specific cell populations. These transcriptomic signatures can guide protein‐level validation and spatial localization within complex and heterogeneous tissues such as the human thymus. Crucially, once protein expression is confirmed, these populations can be prospectively isolated and subjected to functional assays ex vivo, enabling direct assessment of their biological roles.

A defining feature of the polyKRT cell cluster is its expression of ECM components, including Fibronectin‐1 (FN1), which has been implicated in the maintenance of epithelial stemness [123], and ECM‐binding proteins such as basal cell adhesion molecule (BCAM), known to mediate epithelial cell polarization and adhesion to the basal lamina [124]. These molecular features facilitated the spatial localization and quantification of polyKRT cells by 3D‐imaging reconstruction of the basal laminae distribution within the pediatric human thymus. Spatial transcriptomics revealed that polyKRT cells are preferentially localized to subcapsular regions of the cortex and perivascular areas especially of the medulla. First reported in this study, these stem cell niches have been subsequently confirmed by a multimodal spatial analysis [125]. Consistent with the distribution of basal lamina structures, BCAM^+^ΔNTP63α^+^ cells are more abundant in the medulla than in the cortex, as demonstrated by both spatial phenotyping and flow cytometry quantifications.

**FIGURE 4:**
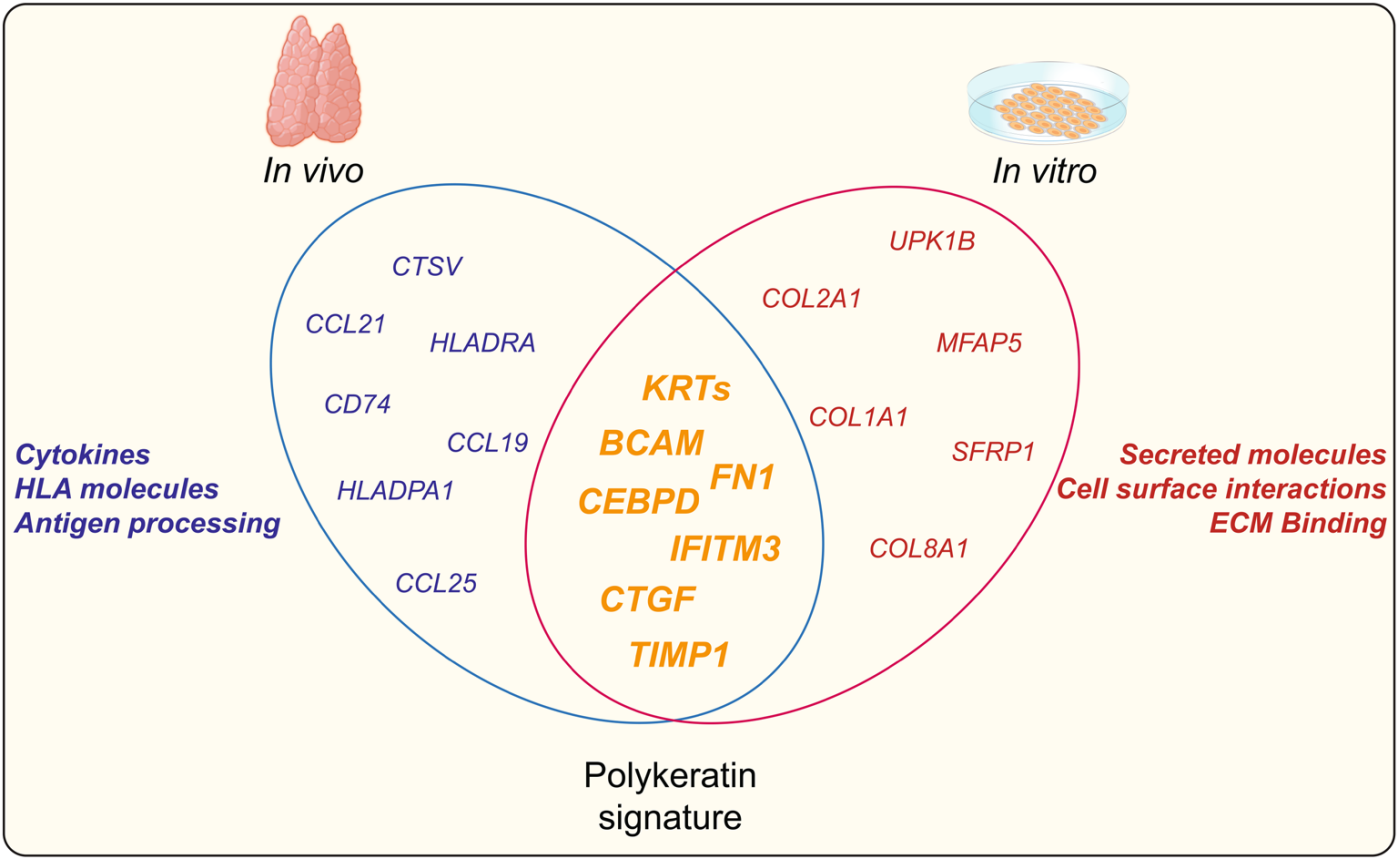
Venn diagram representing transcriptional signature of PolyKRT cells in vivo and in vitro. PolyKRT genes such as keratins, *BCAM, FN1, IFITM3, CEBPD, CTGF*, and *TIMP1* are equally expressed in both conditions. PolyKRT cells in vitro retain their transcriptional signature while upregulating genes associated with proliferation, motility, and extracellular matrix (*COL1A1, COL1A2, UPK1B, MFAP5*, and *SFRP1*) as adaptation to the culture microenvironment; similarly, their in vivo counterpart expresses chemokines and receptors (*CCL19*, *CCL25*, and HLA molecules) important for the crosstalk with thymocytes and the in vivo microenvironment where they reside in specific niches and are in a non‐proliferative status.

Importantly, BCAM serves as a surface marker enabling prospective isolation of polyKRT cells under epithelial stem cell culture conditions. Although comprising a minor fraction of the total epithelial compartment, when isolated by fluorescence‐activated cell sorting (FACS), polyKRT BCAM^+^ cells (which represent < 1% of cTECs and < 5% of mTECs, respectively) were able to give rise to proliferating colonies capable of long‐term self‐renewal in vitro. On the contrary, all BCAM‐negative (> 95% cTECs or 99% of mTECs), although in much higher abundance cannot generate any colony at all. Remarkably, cultured PolyKRT stem cells maintain their core transcriptional identity of their in vivo counterpart, while exhibiting context‐specific adaptations: in the in vitro microenvironment, they are highly activated and proliferating with enrichment in ECM‐associated and proliferative transcripts. They also downregulate functional molecules (e.g., for interactions with thymocytes) compared to when they are in their in vivo niches (Figure 4).

Given the high clonogenic potential and rapid expansion capacity of PolyKRT cells, the holoclone/meroclone/paraclone single‐cell clonal assay was employed to assess the presence of clonogenic TECs with distinct growth potentials and stem‐like phenotypes in vitro. Remarkably, primary cultures of isolated PolyKRT cells revealed heterogeneous colony morphologies, a phenomenon not previously observed in other clonogenic epithelial stem cells, such as skin keratinocytes or airway epithelial cells [85, 97, 126].

When subjected to single‐cell clonal assays and expanded to generate indicator dishes, obtained after 12‐day subculture of one‐quarter of a clonal colony followed by Rhodamine B staining, distinct colony types emerged. Colonies could be classified via phase‐contrast imaging into three morphological types: refractive‐edge (bright cell borders and dim Rhodamine‐B staining), stratified (dark Rhodamine B staining resembling skin keratinocytes), and aborted (characterized by cell pile‐up) [85]. Most importantly, only the refractive‐edge colonies demonstrated long‐term growth potential along with the capacity to generate all three colony types upon further subculture, whereas stratified clones exclusively produced stratified or aborted colonies (Figure 3B).

This degree of clonal heterogeneity has not been previously described in epithelial stem cells, introducing a new level of complexity to the classification of holoclones, meroclones, and paraclones. Additionally, refractive‐edge colonies exhibited an atypical surface marker profile, CD90^+^EpCAM^−^, in contrast to stratified cells, which were CD90^−^EpCAM^+^, consistent with the phenotype of conventional epithelial stem cells such as skin keratinocytes and airway cells. These findings suggest that CD90 expression marks a mesenchymal‐like program in TECs, potentially reflecting the unique 3D architecture of the thymic epithelial network [52, 85, 127].

## 6 Stem Cell Potency and De Novo Functional Reconstitution of a Human Thymus

Embryonic and induced pluripotent stem cells (iPSC) possess the capacity to give rise to any cell type in the body (pluripotency). In contrast, the potency of a somatic progenitor or stem cell refers to its ability to differentiate into one or more specific cell types within a tissue. For instance, inter‐follicular epidermal stem cells are unipotent as they give rise to a single tissue type, the differentiated layers of the epidermis; hair follicle or airway stem cells are multipotent as they generate several distinct cell types within their respective organs [128, 129]. Rodent thymic progenitors and stem cells have also been shown to exhibit multipotency in vivo, and intriguingly, cultured clonal TECs from rats can even demonstrate differentiation potential that extends beyond tissue and germ layer boundaries [26].

But what about the potency of human thymic epithelial stem cells? One of the primary challenges lies in developing robust assays for human cells that can reveal whether a single stem cell can give rise to one or multiple differentiated subtypes, an essential measure of its potency. Yet, defining a cell as a true stem cell goes beyond lineage potential; it must also demonstrate functional differentiation, the ability to generate mature cell types that can restore or reconstruct the organ function. Most of these assays rely on in vivo transplantation chimeric models, whereas recreating the full complexity of organ development in vitro remains a major hurdle. To date, the de novo generation of a fully functional human organ from cultured stem cells in vitro has not been achieved for any organ system, still remaining one of the most ambitious and critical goals of modern regenerative medicine.

The requirement of inductive developmental signals typically provided by embryonic or fetal cells, or at least mesenchymal or other supporting cells, is at the basis of the assays aiming at demonstrating both the potency of thymic epithelial stem or progenitor cells and the functional capabilities of their differentiated progeny as discussed above (Table 2). Nevertheless, the ability to clonally expand human thymic stem cells in vitro provides a unique opportunity to rigorously test the fate potency of individual PolyKRT clones within a controlled microenvironment. This approach allows direct assessment of whether a single stem cell can give rise to multiple thymic epithelial lineages, independent of external cues.

**FIGURE 5:**
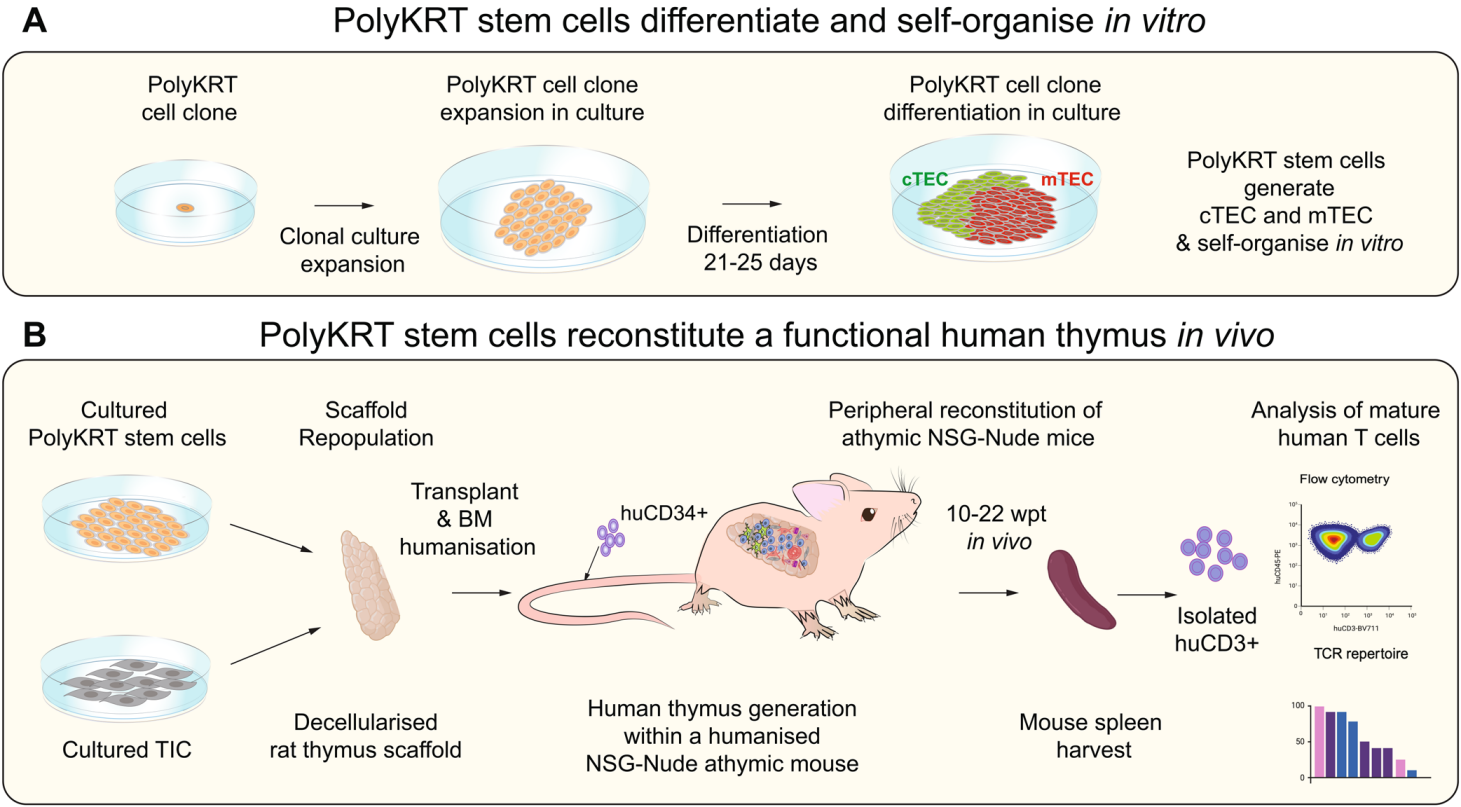
PolyKRT stem cells differentiate and self‐organize in vitro and reconstitute functional cortex and medulla in vivo. Schematics representing in vitro (A) and in vivo (B) assays showing PolyKRT stem cells retain multilineage differentiation capacity and support thymic functionality. (A) PolyKRT single cell clones seeded in vitro with no support from other stromal or hematopoietic cells self‐organize and differentiate into cTEC and mTEC subtypes. (B) PolyKRT stem cells seeded together with interstitial cells within rat thymic acellular thymic matrix repopulated and transplanted in a humanized immunodeficient athymic mouse model (*NSG‐Nude*). At 10–22 weeks post‐transplantation, the progeny of PolyKRT cells differentiate into cTEC and mTEC lineages, upregulate functional markers, and support T cell maturation within the repopulated scaffold. The creation of a functional thymic microenvironment within the scaffold enables reconstitution of the mouse periphery with human naïve CD4^+^ and CD8^+^ T cells.

A key advancement in assessing thymic epithelial stem cell potency has been the development of assays in which only epithelial cells are present, with no contribution from other supporting cell types. In this context, the use of clonal PolyKRT stem cells offers a particularly important means, as it allows direct demonstration of multipotency, that is the capacity of a single stem cell to generate both cortical and medullary thymic epithelial lineages. These clonal cells have been shown to self‐organize and differentiate into major epithelial cortical (cTEC) and medullary (mTEC) subtypes in vitro, even in the absence of external microenvironmental cues or other thymic cell populations [85] (Figure 5A).

This assay similarly to the 3D organoid systems [79] detailed earlier and in Table 2 is powerful for lineage tracing and fate potency assessment. However, they fall short in demonstrating full functional maturation of specific cell subtypes. For example, key molecular markers, such as AIRE, essential for central tolerance, require inductive signals from other cell types, notably RANKL provided by developing thymocytes [79, 130].

Consequently, more complex assay systems incorporating additional cellular components are required to fully evaluate the functional capacity of cultured stem cells, and particularly their ability to reconstitute a thymic microenvironment capable of supporting T cell development, the central function of the thymus as a primary lymphoid organ. One often underestimated yet critical component in organ development and regeneration is the ECM. Beyond providing structural support, the ECM plays a pivotal role in guiding cell migration, activation, and differentiation. In efforts to reconstruct complex organs from scratch, the ECM offers not only the necessary architecture for spatial organization but also the biochemical and biophysical cues required for proper cell–cell communication and differentiation. Over the past 15 years, increasing attention has been given to the use of native ECM scaffolds obtained by perfusion‐decellularization or other methods, which have demonstrated compelling proof‐of‐principle for their capacity to support in vivo organ reconstitution in several fields including the thymus [52, 131, 132, 133, 134, 135].

**FIGURE 6:**
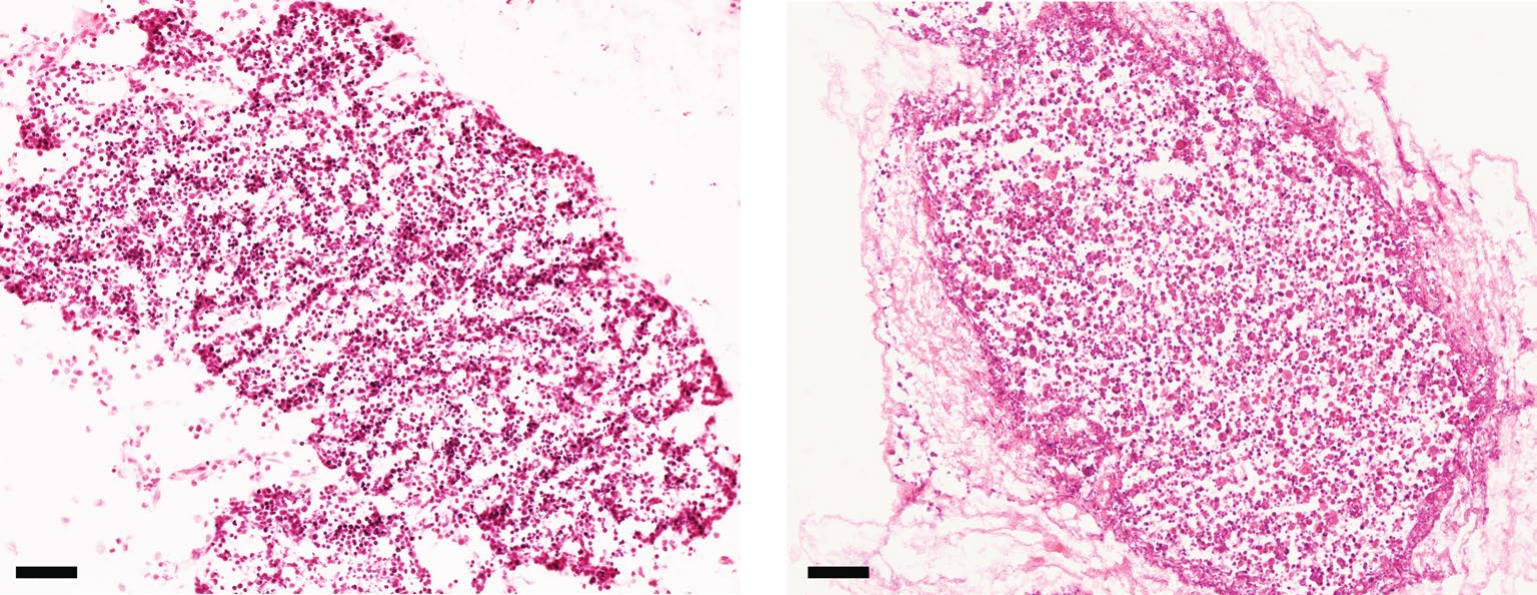
Cultured TECs and TICs seeded within rat thymic extracellular matrix (ECM) recapitulate early morphogenesis of human fetal thymus. Hematoxylin and eosin (H&E) staining of a 9‐week post‐conception (wpc) human thymus (left panel) and of an acellular rat scaffold repopulated with expanded human thymic epithelial and interstitial cells, co‐cultured for 5 days before fixation and histological analysis (right panel). Stromal cells reorganize along the rat ECM with a pattern mimicking the one observed in early human fetal thymus during organ morphogenesis. Scale bars, 50 μm.

A striking demonstration of the ECM's ability to guide and support morphogenesis even ex vivo is the successful repopulation of native acellular thymic scaffolds with cultured human thymic cells. Remarkably, even rodent‐derived ECM has been shown to support human cell morphogenesis in vitro, recapitulating early fetal thymic architecture [52]. As illustrated in Figure 6, co‐seeding human TECs and TICs at a defined ratio results in the reconstitution of a stromal pattern that closely resembles the immature structure of a 9‐week post‐conception human thymus.

Given the ability of cultured human thymic cells to interact with ECM and to recapitulate morphogenetic patterns in vitro, the critical next question was whether such structures could further differentiate and mature to perform the core function of the human thymus. Namely, could they attract human lymphoid progenitors from the bone marrow, organize into functionally distinct cortical and medullary compartments, express key epithelial markers and functional molecules, and ultimately support the maturation of both CD4^+^ and CD8^+^ T cells capable of exiting the thymus, repopulating the peripheral immune system, and producing cytokines when stimulated?

To experimentally approach this, humanized mouse models represent an important tool [136]. In these models, murine HSCs are replaced with human CD34^+^ HSCs derived from cord blood or fetal liver, allowing for the study of human hematopoiesis, immune development, and disease, including leukemia and cancer [137, 138, 139]. However, a major limitation arises when using these models to study thymus biology. The widely used NSG mouse background carries a mutation that impairs HSC differentiation across all lineages, yet retains a rudimentary thymic epithelial anlage. Upon engraftment with human CD34^+^ cells in the bone marrow, this mouse thymic remnant reactivates, producing a limited and delayed wave of T cells. Critically, these T cells develop under the instruction of murine TECs and are restricted to murine MHC, not human, compromising their physiological relevance. Achieving the development of a fully human thymus, with TECs of human origin, therefore, would represent a major breakthrough. It would enable the full reconstitution of a human immune system in a humanized mouse model and would open new experimental and potentially therapeutic avenues (Table 2).

A milestone was achieved in vivo by implanting native thymic ECM repopulated with cultured human epithelial cells and interstitial cells into NSG‐Nude mice. These animals represent a model of particular importance because they are not only immunodeficient but also completely lack the thymic anlage as a result of a *Foxn1* null mutation [52]. Thus, they are fully athymic, ensuring that any thymocytes retrieved from the ectopic graft as well as any human T cells observed in the periphery have developed entirely within the reconstructed human thymic tissue (Figure 5B). Crucially, when PolyKRT stem cells were implanted, they not only interacted with co‐seeded TICs, but also attracted and responded to invading lymphoid progenitors from the humanized bone marrow supporting the development of CD3^+^ thymocytes able to repopulate the periphery of engrafted NSG‐Nude mice. Within the graft, PolyKRT‐derived TECs organized into distinct cortical and medullary compartments, expressed key transcription and specific marker genes essential for thymic function, such as *FOXN1*, *CD274*, LY75, AIRE, and HLA class II, and gave rise to the complex cellular heterogeneity characteristic of a fully developed thymic medulla. This included a progenitor population (*CLDN3/4* ^+^, ASCL1^+^), Hassall's bodies (KRT10^+^), myoid cells (MYOG^+^), ionocytes (KRT7^+^), and neuroendocrine mTECs (SYP^+^, SOX2^+^), as recently reported [85].

The relevance of thymic epithelial stem cells becomes apparent when considering the critical role of peripheral antigen presentation within the thymus. The recently emerging concept that medullary “mimetic” cells [107, 140] mediate AIRE‐independent presentation of tissue‐specific antigens highlights the importance of understanding the cellular origins of this diversity [141]. If such heterogeneity arises from a single progenitor population, PolyKRT stem cells emerge as a primary candidate for investigating the mechanisms that govern epithelial plasticity and distinct cell fate decisions. Regulation of PolyKRT cell differentiation may therefore shape immune tolerance to specific antigens in both physiological and pathological conditions, including autoimmunity.

**FIGURE 7:**
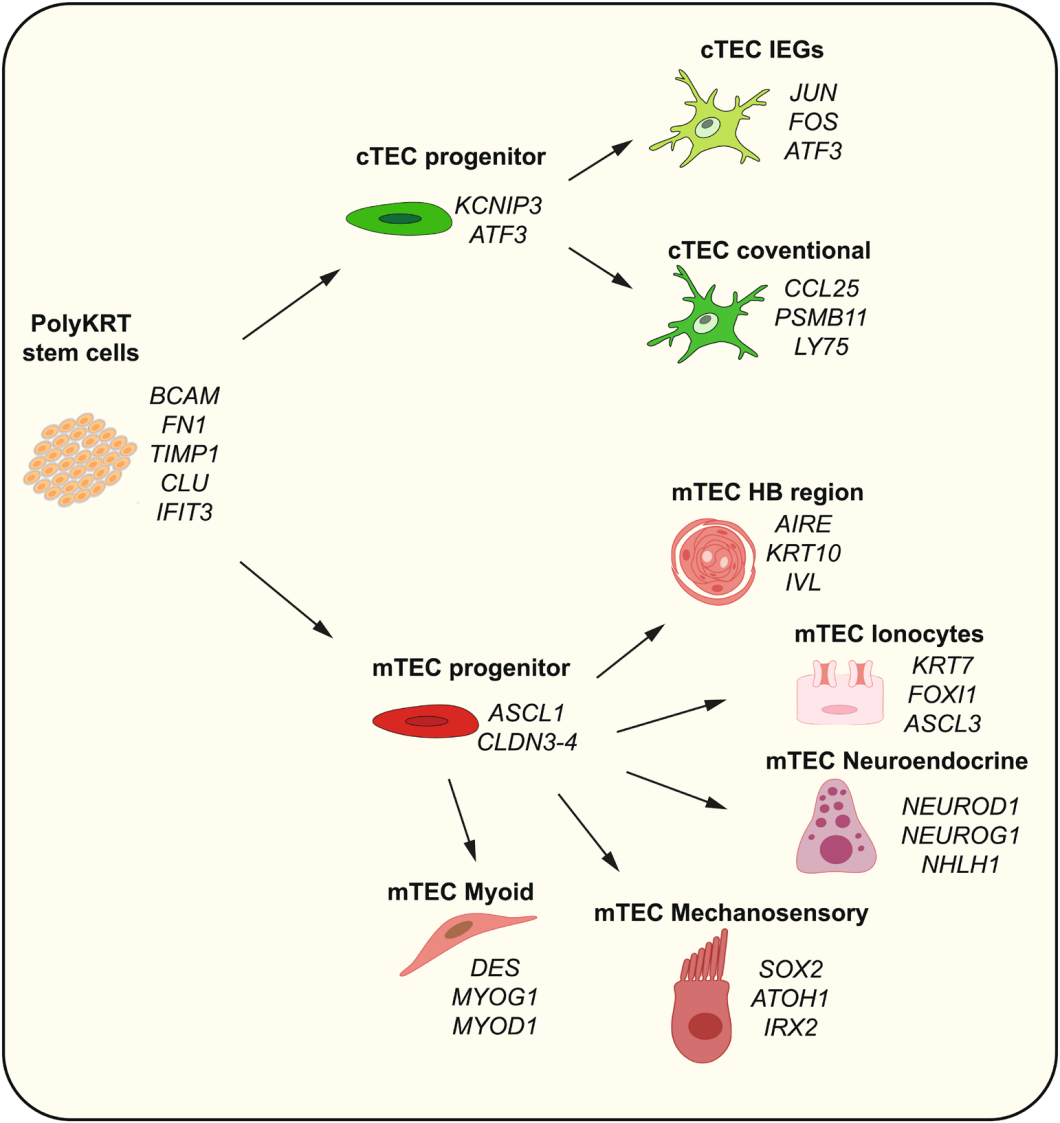
Multilineage differentiation potential of PolyKRT stem cells. PolyKRT stem cells give rise to both cortical and medullary thymic epithelial progenitors. These progenitors transiently upregulate *ATF3* and *KCNIP3* in a shared transition cluster before committing to the cortical (cTEC) lineage. cTEC differentiation results in two main subtypes: one expressing canonical cortical markers (e.g., *PSMB11*) and another co‐expressing immediate early genes (IEGs), suggesting functional specialization. On the medullary lineage (mTEC), progenitors upregulate *ASCL1* and *CLDN* family markers before differentiating into diverse subsets, including myoid, neuroendocrine, mechanosensory, ionocyte, and Hassall's body (HB) region cells. Each subset is defined by distinct gene expression profiles, with representative markers shown in the figure [85].

The PolyKRT cell model thus provides unequivocal evidence of a functional, fully human thymus reconstructed from cultured TECs unraveling their stemness. Beyond demonstrating organ‐level function, it also reveals the intrinsic multilineage potency of human thymic epithelial stem cells and their ability to generate the full spectrum of TEC subtypes, including “mimetic” medullary diverse cell types as illustrated and summarized in the schematic shown in Figure 7.

## 7 Open Questions and Future Directions

Long under‐investigated, the thymus has recently gained renewed attention and growing research interest. Traditionally studied through the lens of developmental immunology, research on the thymus has long focused on its role as an instructive environment for T cell maturation, primarily examining the function of thymic epithelial and DCs in orchestrating thymocyte selection. In murine models, especially, each stage of T cell development has been extensively mapped, with a well‐established framework for understanding positive and negative selection as the basis for generating a self‐tolerant T cell repertoire.

Yet, this classical immunological perspective has proven insufficient to decipher the high complexity of this organ and consequent implications for its function during the human lifespan. Over the past decade, the thymus, with its unique identity as a lympho‐epithelial organ, has become a ground for exploration by stem cell biologists, particularly those with an epithelial biology background. The traditional dichotomy of cortex versus medulla, once central to defining thymic structure and function, now appears overly simplistic. Within the so‐called cortical TEC (cTEC) population, we now recognize a spectrum that includes stem cells, progenitors, and fully differentiated antigen‐presenting cells at various activation states. In the medulla (mTEC), the diversity is even more striking: the compartment harbors myoid, neuroendocrine and mechanosensory cells, ionocytes, and a range of molecular “mimetics” cell types named more by transcriptomic profiles than by clearly defined functional roles. This complexity challenges long‐held assumptions about how negative selection is carried out and calls for a redefinition of thymic architecture in both spatial and functional terms.

In this review, we made the effort to approach the field from the perspective of stem cell and developmental biology, highlighting recent breakthroughs that are beginning to reshape our understanding of thymus biology while recognizing and integrating the historical background. Here, we aim to identify and clarify the fundamental questions that remain unanswered, not only for immunologists but also for stem cell and developmental biologists, as well as biomedical engineers working in regenerative medicine. At the same time, we explore how cutting‐edge technologies and interdisciplinary approaches are opening new avenues for both discovery and future therapeutic innovation if properly integrated with experimental evidence and validation.

A central unsolved question is how embryonic/fetal thymus development differs from postnatal organ homeostasis, particularly in the generation of thymic cell complexity and the organ regenerative capacity driven by progenitor and stem cells. We think that progress has also been limited by the absence of a common nomenclature across immunology, stem cell biology, and developmental biology, hindering agreement on the identity of key cellular players at each stage. Cells isolated from embryonic or fetal organs possess developmental potential intrinsic to organ growth during ontogeny. By contrast, somatic progenitor and stem cells in postnatal and adult stages exhibit properties that are shaped by the specific organ and tissue context, as their function and homeostasis are regulated differently. Here, we aim to clarify the experimental evidence defining thymic epithelial progenitor and stem cells in both mouse and human, and to address the paradox that an involuting organ can retain cells in postnatal life that are capable of expanding in culture and rebuilding organ structure and function.

Therefore, the human thymus is a remarkable yet enigmatic organ, requiring answers to a number of central and still open questions. To address them, we must combine classical experimental methods with new technologies and, ideally, integrate insights from multiple disciplines. In fact, the questions we face are not merely technical. They are fundamental to understanding how this organ shapes immunity across the human lifespan.

Adult life and aging: after the pediatric years, how much regenerative capacity does the thymus truly retain? As it undergoes gradual involution, how do PolyKRT stem cells and their niches adapt to this progressive transformation? When injury occurs, how do they respond, and do their identity, phenotype, and potency change as the years pass?

Role of thymic mesenchyme: within the thymus itself, how do the thymic mesenchymal or interstitial cells contribute to regeneration and support T cell development?

Rebuilding the thymus outside the body: how could stem cells be harnessed to reconstruct a functional thymus in vitro? Could we generate naïve, functional T cells in a physiologic microenvironment outside the body? What are the minimal and sufficient cellular and environmental components required for such a system?

Beyond cellular components: why does the thymus harbor such a rich and complex ECM? What are its physical and biochemical properties? Does it interact directly with developing thymocytes as well as with the inductive and supporting stromal and epithelial cells? If so, could we design biomimetic ECM or advanced hydrogels with fully tuneable properties to replace the native matrix and enable the reconstruction of an entire organ?

Functions beyond immunity: why does the thymus maintain such remarkable cellular complexity and heterogeneity? Could the so‐called mimetic cells keep hidden roles for the thymus that extend beyond conventional immune function?

Modeling human immunity: we have already proof of principle of reconstituted human thymus in humanized mice; can we now create next‐generation models that fully reproduce the human immune system, enabling, for instance, the safe preclinical testing of novel immunotherapies? And could such in vivo platforms, together with organ ex vivo reconstruction strategies, unearth the molecular and functional mechanisms of human immune tolerance?

In summary, understanding how the thymus regenerates across the human lifespan and harnessing this knowledge for thymus organ reconstruction as well as modulation of its function remain important immunological challenges of regenerative medicine. Progress will depend on combining immunology, stem cell biology, biomaterial engineering, and humanized modeling to bring thymic regeneration to clinical applications.

## Author Contributions

R.R.: Data curation, visualization, contributed to writing; P.B.: Conceptualization, visualization, writing the manuscript.

## Funding

P.B. was supported by the European Research Council (ERC Starting Grant no. 639429); the UK Engineering and Physical Sciences Research Council (EPSRC HEU‐Guarantee ERC Advanced Grant EP/U536817/1 and ERC Proof‐of‐Concept EP/Y026543/1); the Rosetrees Trust (grants M362‐F1, M553, CF/100014, and CF‐2023‐M‐2/114); the UK Medical Research Council (MR/Z505808/1); London Advanced Therapies–Research England (C2N‐AT.006); the MRC Confidence in Concept scheme (MC_PC_17180); Innovate UK Smart grants (nos. 10005465 and 10032909); the Crick Idea‐to‐Innovate scheme (P2023‐0004); and the National Institute for Health Research Biomedical Research Centre at Great Ormond Street Hospital for Children NHS Foundation Trust (NIHR GOSH BRC). R.R. was supported by a Marie Skłodowska‐Curie Individual Fellowship (MSCA‐IF no. 896014) and the Rosetrees Trust (CF‐2023‐M‐2/114). This work was supported by the Francis Crick Institute, which receives its core funding from Cancer Research UK (CC0102), the UK Medical Research Council (CC0102), and the Wellcome Trust (CC0102).

## Conflicts of Interest

The authors declare no conflicts of interest.

## Data Availability

The histology data included in this review are available on request from the corresponding author. Other data sharing not applicable to this article as no datasets were generated or analyzed during the current study.
